# Validation of the Alcohol Use Questionnaire (AUQ) in the Italian Context: A Measure for Assessing Alcohol Intake and Binge Drinking

**DOI:** 10.3390/ejihpe15070137

**Published:** 2025-07-17

**Authors:** Eleonora Topino, Alessio Gori

**Affiliations:** 1 Department of Human and Social Sciences, Mercatorum University, Piazza Mattei, 10, 00186 Rome, Italy; eleonora.topino@gmail.com; 2 Department of Health Sciences, University of Florence, Via di San Salvi 12, Pad. 26, 50135 Florence, Italy; 3 Integrated Psychodynamic Psychotherapy Institute (IPPI), Via Ricasoli 32, 50122 Florence, Italy

**Keywords:** alcohol intake, binge drinking, alcohol use disorder, drinking patterns, addiction, substance use disorder

## Abstract

An accurate assessment of alcohol consumption is essential for identifying at-risk individuals and informing prevention and intervention strategies. The present study aimed to validate the Italian version of the Alcohol Use Questionnaire (AUQ), a self-report instrument designed to assess both general alcohol intake and binge drinking patterns. A sample of 378 Italian participants (54.5% female; *M_age_* = 26.76 years, *SD* = 8.44) completed the AUQ along with additional measures assessing binge eating and psychological vulnerabilities related to addiction. Confirmatory factor analysis supported a bifactor model reflecting two distinct but related dimensions: general intake and binge drinking. Network analysis highlighted the central role of perceived frequency of intoxication within the structure of alcohol-related behaviors. Both AUQ indices showed good internal consistency and significant associations with external variables, particularly impulsivity, dissociation, and affect dysregulation, supporting construct validity. The Italian AUQ emerges as a valid and reliable tool for assessing alcohol use patterns and may be useful in both research and clinical practice.

## 1. Introduction

Alcohol consumption is highly prevalent in the general population and, in many cultural contexts, is regarded as a socially sanctioned behavior commonly associated with socialization, celebration, and conviviality (see Sudhinaraset et al. (2016) for a review). Social norms often promote alcohol use by emphasizing its perceived integrative and disinhibitory functions, thereby contributing to the normalization of consumption, even at excessive levels (Studer et al., 2014). However, such cultural permissiveness may obscure the onset of hazardous drinking behaviors and the progression toward problematic patterns of use.

Alcohol Use Disorder (AUD) is defined in the fifth edition of the *Diagnostic and Statistical Manual of Mental Disorders* (DSM-5 and DSM-5-TR) as a problematic pattern of alcohol use leading to clinically significant distress or impairment, characterized by the inability to control consumption despite adverse consequences (American Psychiatric Association, 2013, 2022). A substantial body of research has documented associations between AUD and other psychological disorders, such as mood (McHugh & Weiss, 2019) and anxiety disorders (Torvik et al., 2019). Alcohol misuse has also been linked to cognitive impairments (see Sachdeva et al. (2016) for a review) and to symptom exacerbation in individuals with pre-existing psychopathologies (Rehm et al., 2017). From a physical health perspective, chronic alcohol use is a well-known risk factor for severe medical conditions, including liver disease (e.g., cirrhosis), cardiovascular disorders, neurodegeneration, and various forms of cancer (Shield et al., 2014; World Health Organization, 2018). At the psychosocial level, AUD is frequently associated with significant impairments in romantic and family relationships (Rodriguez et al., 2014), occupational difficulties (Thørrisen et al., 2019), violent or antisocial behaviors (Boden et al., 2012), and safety-compromising behaviors. such as driving under the influence of alcohol (Valen et al., 2019).

Given the clinical and societal relevance of Alcohol Use Disorder (AUD), the scientific research has increasingly focused on identifying key predictors of the condition, with particular emphasis on levels of alcohol intake and binge drinking patterns (Addolorato et al., 2018; Kranzler, 2023; Tuithof et al., 2014). This focus highlights the need for valid and reliable assessment tools capable of capturing both alcohol consumption patterns and their associated risks. An accurate measurement is essential not only for research purposes but also for early identification and targeted intervention in individuals at risk.

### 1.1. The Alcohol Use Questionnaire (AUQ)

The Alcohol Use Questionnaire (AUQ), originally developed by Mehrabian and Russell (1978), was designed to assess habitual alcohol consumption by evaluating the frequency and quantity of wine, beer, and spirits intake, as well as key behavioral indicators, such as drinking speed and episodes of intoxication. The authors proposed a composite Intake Score to reflect regular patterns of alcohol use, highlighting the importance of both amount and pace of consumption in identifying individuals at risk. A subsequent revision by Townshend and Duka (2002) refined the instrument by introducing a second composite index: the Binge Drinking Score. This score captures risky episodic drinking behaviours by combining three specific indicators: the speed of alcohol consumption, the number of times an individual has been drunk in the past six months, and the percentage of drinking occasions that result in intoxication. According to the authors, the Binge Drinking Score reflects a pattern of acute heavy drinking that may not be detected through average intake measures alone, thus offering a more sensitive indicator of behaviors associated with future alcohol dependence and cognitive impairment. This approach shifts the focus from overall consumption to specific behavioral manifestations of binge episodes and has demonstrated validity in distinguishing between social drinkers and those with high-risk profiles (Townshend & Duka, 2002).

### 1.2. The Present Research

Within the international scientific and clinical context dedicated to the assessment of Alcohol Use Disorder, several tools have been developed to evaluate drinking patterns. Some of the most widely used instruments include the Timeline Follow-Back (TLFB; Sobell & Sobell, 1992), which allows for a detailed reconstruction of daily alcohol consumption over a specified period, and the Alcohol Use Disorders Identification Test (AUDIT; Saunders et al., 1993; Babor & Higgins-Biddle, 2001), commonly employed for screening individuals at risk of hazardous or harmful drinking. Although the AUDIT and TLFB are widely used and internationally recognized tools for assessing alcohol use and associated risks, the validation of the Alcohol Use Questionnaire (AUQ) in the Italian context responds to a distinct yet complementary need. Specifically, this measure offers a concise, theory-based measure that captures both general alcohol intake and binge drinking patterns through two separate composite scores. The AUQ has been employed in various international studies (Betka et al., 2018; Herman et al., 2020), including some conducted in Italy (Laghi et al., 2016). However, to the best of the authors’ knowledge, no published research has systematically evaluated the psychometric properties of its Italian version. Given the growing relevance of accurate alcohol use assessment tools in both the research and clinical settings, the present study aimed to translate and validate the AUQ (Mehrabian & Russell, 1978; Townshend & Duka, 2002) for use in the Italian context.

Building upon the theoretical foundations of the original instrument, the general objective of the present study was to examine the psychometric properties of the Italian version of the Alcohol Use Questionnaire (AUQ; Mehrabian & Russell, 1978; Townshend & Duka, 2002), with a specific focus on the validity of its composite scoring system.

More specifically, this study pursued the following aims:

1. To test the factor structure of the AUQ by conducting a Confirmatory Factor Analysis (CFA), in order to examine the adequacy of a bifactor model reflecting both general alcohol intake and binge drinking patterns.
2. To further investigate the internal structure of the questionnaire through a network analysis approach, allowing for the exploration of the interconnections between items and the identification of the most central elements in sustaining the structure of alcohol-related behaviors.

## 2. Materials and Methods

### 2.1. Participants

The sample consisted of 378 Italian participants (54.5% female; 45.5% male), all of whom reported consuming alcohol (see Table 1).

Their mean age was 26.76 years (SD = 8.44). Most participants reported being single (76.5%), followed by those cohabiting (14.8%) or married (7.7%). Regarding educational background, more than half held a high-school diploma (50.8%), while 25.9% had completed a university degree and 14% a master’s degree. In terms of employment status, the majority were students (63.8%) or employees (19.0%).

### 2.2. Procedure and Ethics

The Alcohol Use Questionnaire (AUQ; Mehrabian & Russell, 1978) was translated into Italian using a standardized back-translation procedure, in line with internationally recognized guidelines (Beaton et al., 2000). First, the original English version of the AUQ was translated into Italian by an independent bilingual translator. This preliminary version was then back-translated into English by a native English speaker with advanced proficiency in Italian. Discrepancies between the original and the back-translated versions were carefully examined and discussed in depth by the research team, with particular attention to preserving not only semantic accuracy but also conceptual and contextual equivalence. This iterative process continued until full agreement was reached on all items, ensuring that the Italian version faithfully reflected the theoretical underpinnings and clinical intent of the original instrument. Participants were recruited online through a snowball sampling procedure, starting from the authors’ social and professional networks. The survey was administered via the Google Forms platform. Before beginning the questionnaire, participants were informed about the general purpose of this study and provided their informed consent electronically. Inclusion criteria were as follows: (a) being at least 18 years old and (b) self-reporting alcohol use. All data were collected anonymously, and no compensation was provided for participation. This study was approved by the institutional Ethics Committee of one of the researchers.

### 2.3. Measures

#### 2.3.1. Alcohol Use Questionnaire (AUQ)

The Alcohol Use Questionnaire (AUQ; Mehrabian & Russell, 1978) is a 12-item self-report measure designed to explore patterns of alcohol consumption over the past six months. It includes items assessing the frequency of drinking, average quantity of alcohol consumed across different beverage types (wine, beer, and spirits), and behaviors indicative of risky drinking, such as speed of alcohol intake (e.g., “How fast do you drink?”), number of times getting drunk, and percentage of times getting drunk while drinking. Of the 12 items, 6 contribute to the calculation of an Intake Score, which captures the overall quantity and regularity of alcohol consumption. The Intake Score is computed using the following weighted formula:

Intake Score = item 3 + item 6 + item 9 + (4 × item 10) + item 11 + (0.2 × item 12)

In addition, a Binge Drinking Score (BS) can be calculated following the procedure suggested by Townshend and Duka (2002), by combining the 3 items related to binge-like drinking patterns: speed of drinking (item 10), number of times being drunk in the past six months (item 11), and percentage of times getting drunk while drinking (item 12). The formula used is:

Binge Drinking Score = 4 × item 10 + item 11 + 0.2 × item 12

#### 2.3.2. Binge Eating Scale (BES)

The Binge Eating Scale (BES; Gormally et al., 1982; Italian version: Di Bernardo et al., 1998) is a self-report measure designed to assess the presence and severity of binge eating behaviors. It consists of 16 items featuring multiple response options with 3 or 4 statements of increasing severity, from which respondents select the one that best describes their experience. Higher scores indicate a greater severity of binge eating symptomatology. In the present sample, the BES showed excellent internal reliability (ω = 0.92).

#### 2.3.3. Seven Domains Addiction Scale (7DAS)

The Seven Domains Addiction Scale (7DAS; Caretti et al., 2018) is a self-report measure designed to assess seven core psychological domains commonly implicated in Substance-Related and Addictive Disorders. The scale is part of the broader Addictive Behavior Questionnaire (ABQ; Caretti et al., 2018) and is composed of 49 items, grouped into seven subscales: separation anxiety, affect dysregulation, somatoform and psychological dissociation, childhood traumatic experiences, impulse dyscontrol, compulsive behavior and ritualization, and obsessive thoughts. Each subscale includes 7 items rated on a 5-point Likert scale, with higher scores indicating a higher presence of the corresponding psychological trait or vulnerability. In the present study, the 7DAS subscales showed good internal reliability (McDonald ω ranging from 0.78 for compulsive behavior and ritualization, to 0.89 for separation anxiety and obsessive thoughts).

### 2.4. Data Analysis

Data were analyzed using the SPSS (v. 28.0; IBM, New York, NY, USA), AMOS (v. 24.0; IBM, New York, NY, USA), and JASP (v. 0.19.3; JASP Team, 2024). Within the dataset, no missing responses were observed, as the online survey platform was configured to enforce complete item response prior to submission. Consequently, no imputation methods or case deletion procedures were required. Descriptive statistics were first calculated to explore response distributions for each item of the Alcohol Use Questionnaire (AUQ), providing information on the frequency and quantity of alcohol consumption, as well as indicators of risky drinking behaviors (e.g., speed of drinking, number of times drunk, and percentage of times getting drunk while drinking). Subsequently, a series of Confirmatory Factor Analyses (CFAs) were conducted to test the latent structure of the AUQ. In line with the theoretical framework and scoring system proposed by the original authors (Mehrabian & Russell, 1978) and further elaborated by Townshend and Duka (2002), a bifactor model was specified to simultaneously account for the Intake Score and the Binge Drinking Score. To preserve the theoretical weighting of the composite scores, item 10 (drinking speed) and item 12 (percentage of times drunk) were entered into the model after being multiplied by 4 and 0.2, respectively, as outlined in the original scoring algorithm. The Binge Drinking Score was modeled as a specific factor indicated by item 10 (weighted), item 11 (raw), and item 12 (weighted), while the Intake factor included all 6 items contributing to the composite intake score. Model fit was evaluated based on multiple indices. The chi-square divided by degrees of freedom (χ^2^/df) was considered acceptable when less than 5 (Marsh & Hocevar, 1985). The Comparative Fit Index (CFI), Tucker–Lewis Index (TLI), and Normed Fit Index (NFI) were interpreted as indicators of good fit if values exceeded 0.90 (Hu & Bentler, 1999; Kline, 2015). The Root Mean Square Error of Approximation (RMSEA) was interpreted in line with the standard guidelines, with values below 0.08 indicating an acceptable model fit (Marsh et al., 2004). The 90% confidence interval of the RMSEA was also examined, as it reflects the precision and potential variability of the estimate (MacCallum et al., 1996). Intervals entirely below 0.08 are typically considered indicative of a satisfactory fit, whereas upper bounds exceeding 0.10 may signal potential issues with model adequacy. Furthermore, the p-close statistic was used to assess whether the RMSEA value supported the hypothesis of a close fit (defined as RMSEA ≤ 0.05); values above 0.05 suggest that the model provides a sufficiently close approximation to the population data (Chen et al., 2008). The Standardized Root Mean Square Residual (SRMR) was interpreted as indicating a good fit when below 0.08 (Hooper et al., 2008). In order to evaluate the added value of the bifactorial structure compared to a more parsimonious model, a unifactorial solution was also tested through CFA. The two models were compared by calculating the chi-square difference test (Δχ^2^), which assesses whether the increase in model complexity significantly improves the model fit. A statistically significant Δχ^2^ (*p* < 0.05) is considered indicative of a better fit of the more complex model to the data (Byrne, 2020; Jackson et al., 2009; Kock et al., 2021). To assess the internal consistency of the scales under investigation, McDonald’s omega (ω; McDonald, 1999) was computed, along with its 95% confidence interval. This coefficient is considered a particularly accurate and flexible estimate of reliability in the presence of multidimensional constructs or items with non-uniform factor loadings. In line with the existing recommendations (Brunner et al., 2012), values of *ω* ≥ 0.70 were considered indicative of acceptable reliability. Confidence intervals that lie entirely above 0.70 are generally interpreted as evidence of acceptable reliability (Furr, 2022; Revelle & Zinbarg, 2009). In addition, corrected item-total correlations were examined to evaluate the contribution of each item to the overall coherence of the scale. Correlations ≥ 0.30 were considered acceptable (Nunnally & Bernstein, 1994; Streiner, 2003), while values between 0.20 and 0.30 were considered tolerable when supported by a theoretical justification (DeVellis & Thorpe, 2021). To further inform this evaluation, McDonald’s omega was also recalculated under item deletion for items within this borderline range. Following recommendations from the psychometric literature, only increases in reliability that resulted in a shift across interpretive thresholds (e.g., from <0.70, considered acceptable, to <0.80, considered good; George & Mallery, 2003; Furr, 2022; Kline, 2013) were deemed relevant, provided that the increase was also practically meaningful (Δ*ω* > 0.03; Streiner, 2003; Furr, 2022). The co-occurrence of these two conditions was considered as an indication for potential item removal, particularly when the item also exhibited marginal theoretical relevance (DeVellis & Thorpe, 2021; Clark & Watson, 2019). To further explore the internal structure of the Alcohol Use Questionnaire and the relationships among its core items, a network analysis was conducted. A regularized partial correlation network (also known as a Gaussian Graphical Model) was estimated using the EBICglasso procedure (Friedman et al., 2008), which applies the graphical Least Absolute Shrinkage and Selection Operator (GLASSO) regularization based on the Extended Bayesian Information Criterion (EBIC). The tuning parameter *γ* was set to 0.5, as recommended in the literature (Foygel & Drton, 2010), to balance sensitivity and specificity in edge selection and avoid overfitting. The network consists of nodes (the observed items) and edges (the conditional relationships between items, controlled for all other items in the network). Following Ferguson (2021), edge weights ≤0.20 were interpreted as small, values between >0.20 and ≤0.50 as moderate, and values >0.50 as large. The expected influence index was used to estimate node centrality (Robinaugh et al., 2016; Bringmann et al., 2019; Epskamp et al., 2018), as it is more appropriate than traditional centrality measures when all nodes contribute with different weights. This allows for a more accurate identification of the items that play a greater role in sustaining the network structure. Finally, to examine the associations between the AUQ scores (both intake and binge drinking) and the variables included to assess divergent validity, Pearson’s correlation coefficients were calculated.

## 3. Results

As reported in Table 2, 56.6% of participants drank wine on 1–2 days per week, and 50.5% drank beer with the same frequency. Spirits were consumed on 1–2 days per week by 59.0% of the sample. Most participants reported drinking between 0.5 and 3 units per drinking day across beverage types. Concerning risky drinking behaviors, 40.2% reported a drinking speed of two or more drinks per hour, and 56.9% reported having been drunk at least once in the past six months.

### 3.1. Factor Structure and Internal Consistency

**Figure 1:**
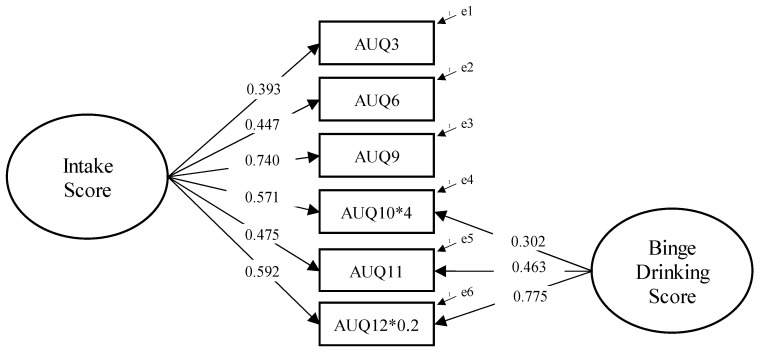
Confirmatory Factor Analysis for the bifactor model of the AUQ. *Note:* AUQ10*4 and AUQ12*0.2 indicate that items 10 and 12 were multiplied by 4 and 0.2, respectively, in accordance with the original scoring algorithm proposed by Townshend and Duka (2002), to preserve the theoretical weighting of the composite scores.

The bifactor model (see Figure 1), reflecting the theoretical scoring structure of the Alcohol Use Questionnaire (AUQ), showed an overall good fit to the data: χ^2^/df = 2.937, NFI = 0.969, TLI = 0.948, CFI = 0.979, RMSEA = 0.072 (90% CI [0.034, 0.112], p-close = 0.151), SRMR = 0.031. In contrast, the unifactorial solution presented poorer fit indices: χ^2^/df = 6.600, NFI = 0.896, TLI = 0.848, CFI = 0.909, RMSEA = 0.122 (90% CI [0.093, 0.152], p-close < 0.001), SRMR = 0.063. The chi-square difference test confirmed the statistical superiority of the bifactor model over the unifactorial solution (Δχ^2^ = 41.777, Δdf = 3, *p* < 0.001).

Concerning internal consistency, McDonald’s omega for the AUQ Intake Score was good (ω = 0.77; 95% CI [0.73, 0.81]). Item-total correlations ranged from 0.23 (item 3: glasses of wine per week) to 0.69 (item 12: % of times drunk when drinking), indicating that most items contributed moderately to strongly to the overall scale. Item 3 showed the weakest correlation and was the only item below the commonly accepted threshold of 0.30. However, its removal would have resulted in only a slight increase in omega (from 0.77 to 0.78), which was not deemed substantial. Therefore, the item was retained to preserve content validity and ensure comprehensive coverage of alcohol intake behaviors. Furthermore, McDonald’s omega for the AUQ Binge Drinking Score was acceptable (*ω* = 0.75; 95% CI [0.71, 0.80]). All item-total correlations exceeded the commonly accepted threshold of 0.30, indicating adequate internal coherence. Specifically, the lowest value was 0.51 for item 10 (drinking speed), while the highest was 0.71 for item 12 (% of times drunk when drinking), and item 11 (times drunk in the last 6 months) showed an intermediate correlation of 0.61.

### 3.2. Network Analysis

**Figure 2:**
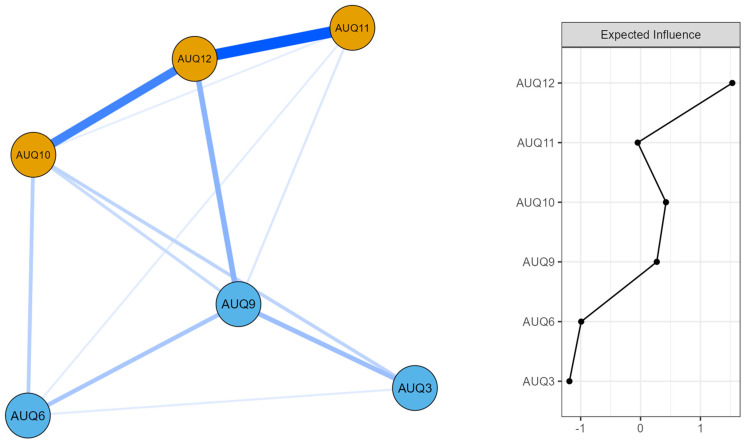
Network structure and expected influence centrality of the AUQ items. *Note:* The left panel displays the network structure estimated from the AUQ items. Thicker and more saturated edges represent stronger associations. Nodes in orange represent items specifically related to the Binge Drinking Score. The right panel shows the expected influence centrality index for each item, indicating their relative importance within the network. AUQ3 = Glasses of wine per week; AUQ6 = Pints of beer per week; AUQ9 = Shots of spirits per week; AUQ10 = Drinking speed; AUQ11 = Times drunk in last 6 months; AUQ12 = % of times drunk when drinking.

The network structure of the Alcohol Use Questionnaire (AUQ) items related to binge drinking included six nodes and 13 non-zero edges (see Figure 2).

Overall, the strongest associations emerged between AUQ6 (pints of beer per day) and AUQ9 (shots of spirits per day; edge weight = 0.480), followed by the connection between AUQ3 (glasses of wine per day) and AUQ9 (edge weight = 0.358), and between AUQ9 and AUQ12 (percentage of times drunk when drinking; edge weight = 0.218). Regarding node centrality, AUQ12 showed the highest expected influence.

### 3.3. Pearson’s Correlation

Pearson’s correlation showed significant associations between the two AUQ indices (Intake Score and Binge Drinking Score) and the variables considered for construct validity (see Table 3). Both indices were significantly and positively associated with the Binge Eating Scale (*r* = 0.300, *p* < 0.01 for intake; *r* = 0.351, *p* < 0.01 for binge drinking). Moreover, significant correlations emerged between both AUQ indices and six out of the seven 7DAS domains. The strongest associations emerged with Impulse Dyscontrol (r = 0.262 and r = 0.291) and Somatoform and Psychological Dissociation (r = 0.228 and r = 0.232). Weaker but significant associations were also found with Separation Anxiety, Affect Dysregulation, Compulsive Behavior and Ritualization, and Obsessive Thoughts. No significant correlations were found with the Childhood Traumatic Experiences domain.

## 4. Discussion

Alcohol intake and binge drinking patterns are widely recognized in the scientific literature as key risk factors for the development and maintenance of AUD (Addolorato et al., 2018; Kranzler, 2023; Tuithof et al., 2014). This emphasis underscores the necessity of employing valid and reliable assessment measures to capture both habitual consumption and risky episodic drinking. In light of this, the present study aimed to test the psychometric properties of the Italian version of the Alcohol Use Questionnaire (AUQ; Mehrabian & Russell, 1978; Townshend & Duka, 2002), to support its use in both the research contexts and clinical settings.

### 4.1. Factor Structure

The Confirmatory Factor Analysis (CFA) supported the statistical adequacy of a bifactor model representing two core dimensions of alcohol use: intake and binge drinking. This structure is consistent with the theoretical foundations laid out by both the original authors of the Alcohol Use Questionnaire (Mehrabian & Russell, 1978) and the subsequent conceptual refinements introduced by Townshend and Duka (2002). Specifically, the model allowed for the computation of two composite scores (i.e., Intake and Binge Drinking) based on the original weighting system and yielded a good overall fit to the data across all the main indices. Both composite scores demonstrated satisfactory internal consistency, with McDonald’s omega coefficients exceeding the recommended threshold (Brunner et al., 2012; McDonald, 1999), further supporting the reliability of the two subdimensions. Notably, when compared to a unifactorial alternative, the bifactor model showed a significantly better fit. This result empirically supports the added value of modeling binge drinking as a specific dimension within the broader construct of alcohol intake (Kuntsche et al., 2017). Although the Binge Drinking Score is derived from a subset of the intake items, its separate representation in the bifactor structure allows for the identification of distinct patterns of episodic risky drinking, which may not be fully captured by aggregate measures of frequency and quantity alone. This differentiation is also consistent with the previous research supporting the need to distinguish between average consumption over time and amount consumed on a single occasion (see Dawson and Room (2000) for an overview). Such a distinction can provide more clinically nuanced information about alcohol use behavior, particularly in identifying individuals at risk for escalation or adverse consequences.

### 4.2. Network Structure and Centrality Patterns

Rather than assuming a latent common cause, the network analysis approach conceptualizes items as components of a complex system of mutual interactions, where each element (node) may influence and be influenced by the others within the network (Borsboom & Cramer, 2013).

In the present study, the resulting network consisted of six nodes and thirteen non-zero edges, reflecting a well-connected system of associations between key indicators of alcohol consumption patterns (see Figure 2). Among the strongest connections, the edge between the number of pints of beer consumed per drinking day (AUQ6) and the number of shots of spirits (AUQ9) emerged as the most substantial, suggesting that a higher consumption of one type of beverage is strongly associated with an elevated intake of another. Notably, AUQ9 was also moderately associated with the number of glasses of wine consumed (AUQ3), and with the percentage of drinking occasions that led to intoxication (AUQ12). These results point to AUQ9 as a bridge node connecting different types of alcohol intake with outcome-related behaviors, such as the likelihood of getting drunk. This pattern may reflect a shift in drinking motivation among individuals with more problematic alcohol use: rather than being driven by taste preferences for specific beverages, their consumption appears to be guided by a more indiscriminate pursuit of alcohol per se, often aimed at achieving intoxication. This interpretation aligns with motivational models of alcohol use (Cox & Klinger, 1988) and with neurobiological perspectives that highlight how, in the context of addiction, the consumption of alcohol becomes increasingly detached from specific sensory or social cues, and more tightly linked to compulsive reward-seeking processes (Koob & Volkow, 2016). This was further supported by the centrality analyses based on Expected Influence (Robinaugh et al., 2016; Bringmann et al., 2019). In particular, item 12 (“*What percentage of times you drink do you get drunk*?”) emerged as the most central node in the network, showing the highest expected influence score. This finding highlights the core role of perceived frequency of intoxication in sustaining the overall pattern of alcohol-related behaviors. Such data are in line with previous evidence showing that the search for an acute intoxication state may be a key motivator behind excessive and problematic alcohol use (Winograd et al., 2016), and that alcohol intoxication is significantly associated with a wide range of alcohol-related problems (Neal & Carey, 2007). This finding highlights AUQ12 as a potential clinically relevant marker for screening purposes.

### 4.3. Associations Between the AUQ Indices and Related Psychological Dimensions

To further explore the construct validity of the Alcohol Use Questionnaire (AUQ), the relationships between the Intake and Binge Drinking Scores and a set of external variables commonly associated with addictive behaviors were examined. The results indicate that both AUQ indices are significantly and positively correlated with binge eating symptoms. In this regard, there is growing evidence of a strong association between alcohol misuse and eating disorders, particularly binge eating (see Azevedo et al., 2021 for a review). The stronger correlation observed between the Binge Drinking Score and binge eating symptoms, compared to the Intake Score, suggests that episodic patterns of excessive alcohol consumption may be more closely aligned with dysregulated eating behaviors, potentially reflecting a common underlying tendency toward loss of control. Consistently, previous studies have shown that binge drinking may exacerbate binge eating behaviors, and vice versa, creating a vicious cycle of dysfunctional patterns (see Escrivá-Martínez et al., 2020 for a review).

In line with this interpretation, both AUQ indices were significantly associated with six out of the seven domains assessed by the Seven Domains Addiction Scale (7DAS), further supporting the link between alcohol-related behaviors and core psychological vulnerabilities implicated in addictive disorders (Caretti et al., 2018). Notably, the strongest correlations emerged with Impulse Dyscontrol and Somatoform and Psychological Dissociation. These findings are consistent with a robust body of literature identifying impulsivity as a central psychological factor in the development and maintenance of alcohol-related problems (Dick et al., 2010; De Wit, 2009). Impulsivity, defined as the tendency to act rapidly without sufficient forethought, has been consistently linked to both increased frequency of alcohol consumption and a heightened likelihood of engaging in binge drinking episodes (see Stautz & Cooper, 2013 for a review). Moreover, a growing body of evidence suggests that addictive behaviors may function as dissociative strategies to escape from internally dysregulated emotional states (Gori et al., 2023a, 2023b). This mechanism appears to be relevant not only in the context of alcohol and substance use (Craparo et al., 2014), but also across various forms of behavioral addictions (Gori et al., 2022; Gori & Topino, 2024; Topino et al., 2024). In line with this, weaker but significant correlations also emerged with Affect Dysregulation, Compulsive Behavior and Ritualization, Separation Anxiety, and Obsessive Thoughts, suggesting a broad pattern of psychological dysregulation associated with higher AUQ scores. Focusing first on Affect Dysregulation and Separation Anxiety, previous research has consistently linked emotional instability and insecure attachment to increased risk of alcohol misuse (Berking et al., 2011; Schäfer & Najavits, 2007; Thorberg & Lyvers, 2006). Individuals with poor emotion regulation skills may use alcohol to manage negative affective states or emotional overload (Weiss et al., 2015; Fox et al., 2008). This is especially true for binge drinking, which is often triggered by acute stress and serves as an immediate form of relief (Kassel et al., 2003). At the same time, separation anxiety (characterized by excessive fear of abandonment) has been associated with heightened emotional reactivity and maladaptive coping, including substance use, particularly in the context of relational distress (Schindler et al., 2005; Thorberg & Lyvers, 2010; Topino et al., 2025). These findings suggest that emotional and interpersonal dysregulation may jointly contribute to risky alcohol consumption patterns. Moreover, the significant correlations between both AUQ indices the domains of Compulsive Behavior and Ritualization and Obsessive Thoughts suggested that repetitive, intrusive, and rigidity-prone cognitive and behavioral patterns may also contribute to alcohol misuse. Compulsivity has been increasingly recognized as a key component in the shift from voluntary to habitual substance use, particularly when consumption becomes ritualized and disconnected from initial motivations (Everitt & Robbins, 2016; Fineberg et al., 2014). From this perspective, alcohol use may follow rigid behavioral scripts aimed at preventing discomfort or regulating tension, even in the absence of strong craving or pleasure. Similarly, the presence of obsessive thoughts has been associated with a greater risk of substance use as a maladaptive strategy to reduce cognitive dissonance or mental distress (Tavares et al., 2003; Robbins et al., 2012). These associations may reflect shared mechanisms between substance-related and obsessive-compulsive spectrum disorders, such as impaired top-down control and cognitive inflexibility (Volkow et al., 2010). The current findings reinforce the importance of addressing cognitive rigidity and compulsive tendencies in the assessment and treatment of problematic alcohol use.

## 5. Limitations and Suggestions for Future Research

The present study had some limitations that should be acknowledged. First, the use of a non-probabilistic, snowball sampling procedure (relying primarily on the authors’ social and professional networks) may have introduced selection bias and limited the generalizability of the findings across different Italian regions and demographic strata. Future research should aim to replicate these results using probabilistic sampling methods or more diverse populations to improve external validity. Consistently, this study relied exclusively on a non-clinical sample, primarily composed of university students. Although this population is particularly relevant for the assessment of alcohol-related behaviors, especially binge drinking, which is common among young adults (Kuntsche et al., 2017), the findings cannot be generalized to clinical populations or individuals with diagnosed psychiatric conditions. Future research should include clinical samples to evaluate the utility of the AUQ in identifying more severe patterns of alcohol misuse. An additional consideration concerns the application of item weightings in the Confirmatory Factor Analysis (CFA), adopted to preserve the original scoring structure of the AUQ. Although this approach ensured consistency with the theoretical and practical use of the instrument, it deviates from conventional CFA practices. Future studies may consider alternative modeling strategies, such as latent variable estimation without weighting, or the use of item parceling to further examine the robustness and generalizability of the factor structure. Moreover, although the network analysis provided useful insights into the internal structure of alcohol-related behaviors, certain methodological limitations should be acknowledged. Specifically, the absence of bootstrapped confidence intervals limits the ability to evaluate the accuracy and robustness of the network structure. Future studies should implement these procedures to ensure more robust and replicable network estimates. Another limitation relates to the absence of both test–retest reliability and criterion validity. Without repeated measurements over time, the temporal stability of the AUQ scores remains unverified. Similarly, this study did not include direct comparisons with gold-standard instruments, such as the AUDIT (Saunders et al., 1993; Babor & Higgins-Biddle, 2001) or the TLFB (Sobell & Sobell, 1992), which would have allowed for a more thorough assessment of concurrent criterion validity. Future research should address these aspects to strengthen the longitudinal robustness and external positioning of the AUQ within the broader framework of alcohol use assessment tools. In addition, measurement invariance across relevant subgroups (e.g., gender, age, and educational level) was not assessed. This limits the ability to determine whether the bifactor structure of the AUQ functions equivalently across different segments of the population. Future studies should address this aspect to verify the structural stability and comparability of the instrument across diverse demographic groups. Finally, all data were based on self-report measures, which are subject to biases, such as social desirability and recall errors. Future studies could benefit from integrating objective indicators of alcohol use, such as behavioral tracking or biological markers, to enhance data validity.

## 6. Conclusions

The present study provides initial evidence for the validity and reliability of the Italian version of the Alcohol Use Questionnaire (AUQ; see Appendix A), supporting its use as a brief and theory-driven measure for assessing both general alcohol intake and binge drinking patterns. The bifactor structure, the network analysis, and the associations with relevant psychological variables all converge in highlighting the instrument’s capacity to capture distinct and clinically meaningful dimensions of alcohol-related behavior. Beyond its psychometric soundness, the AUQ may have practical implications for both research and clinical practice. By providing separate indicators of general alcohol intake and binge drinking, it offers a nuanced assessment of drinking behaviors that can support early detection of at-risk patterns. Indeed, in clinical and primary care settings, brief self-report tools have demonstrated utility in screening for hazardous and harmful drinking (Aertgeerts et al., 2001; Babor & Higgins-Biddle, 2001), and the AUQ may represent a valuable addition in this regard due to its focused measurement of both consumption frequency and risky episodic drinking. The dual scoring system of the AUQ allows practitioners to evaluate both individuals with regular intake and those who engage in binge drinking, patterns often associated with a greater risk for psychological and physical health consequences (Kuntsche et al., 2017; Kranzler, 2023). This distinction is especially relevant in young adult and university populations, where episodic heavy drinking may not be reflected by average intake scores alone but can signal early-stage trajectories toward Alcohol Use Disorder (Addolorato et al., 2018; Winograd et al., 2016). The AUQ can provide valuable information about individual drinking patterns, enabling practitioners to obtain a more detailed picture of alcohol use habits. Furthermore, the questionnaire may serve as a useful tool for monitoring changes in consumption over time, particularly in the context of repeated assessments aimed at evaluating the effectiveness of preventive or therapeutic interventions. The ability to detect these patterns through a concise and structured instrument can inform tailored interventions, brief motivational feedback, or referrals to specialized services. To support its broader implementation, future research should focus on the collection of normative data to facilitate score interpretation at the population level, as well as on the development of digital self-administration platforms. These steps would enhance the accessibility, scalability, and integration of the AUQ into routine screening workflows across clinical, educational, and community-based settings.

## Figures and Tables

**Table 1:** Demographic characteristics of the sample (*N* = 378).

Characteristics		*M* ± *SD*	*N* (*%*)
Age		26.76 ± 8.441	
Sex			
	*Females*		206 (54.5%)
	*Males*		172 (45.5%)
Marital Status			
	*Single*		289 (76.5%)
	*Cohabiting*		56 (14.8%)
	*Married*		29 (7.7%)
	*Separated or Divorced*		3 (0.8%)
	*Widowed*		1 (0.3%)
Education			
	*Middle School diploma*		18 (4.8%)
	*High School diploma*		192 (50.8%)
	*University degree*		98 (25.9%)
	*Master’s degree*		53 (14.0%)
	*Post-lauream specialization*		17 (4.5%)
Occupation			
	*Artisan*		8 (2.1%)
	*Employee*		72 (19.0%)
	*Entrepreneur*		11 (2.9%)
	*Freelance*		13 (3.4%)
	*Manager*		4 (1.1%)
	*Student*		241 (63.8%)
	*Trader*		4 (1.1%)
	*Unemployed*		25 (6.6%)

**Table 2:** Descriptive statistics for AUQ items on alcohol consumption.

Item	Response Category	*N* (%)	Item	Response Category	*N* (%)	Item	Response Category	*N* (%)
1. Wine drinking frequency (days/week)	0 days	81 (21.4%)	2. Glasses of wine per drinking day	0	0 (9.9%)	3. Glasses of wine per week	0	69 (18.3%)
	1–2 days	214 (56.6%)		0.5–1	0.5–1 (29.6%)		0.5–2	144 (38.1%)
	3–4 days	54 (14.3%)		1.5–3	1.5–3 (48.7%)		3–5	95 (25.1%)
	5–7 days	29 (7.7%)		>3	>3 (11.8%)		6–10	53 (14.0%)
	-	-		-	-		>10	15 (4.0%)
4. Beer drinking frequency (days/week)	0 days	148 (39.2%)	5. Pints of beer per drinking day	0	131 (34.7%)	6. Pints of beer per week	0	143 (37.8%)
	1–2 days	191 (50.5%)		0.5–1	160 (42.3%)		0.5–2	162 (42.9%)
	3–4 days	29 (7.7%)		2–3	71 (18.8%)		3–5	54 (14.3%)
	5–7 days	10 (2.6%)		>3	16 (4.2%)		6–10	16 (4.2%)
	-	-		-	-		>10	3 (0.8%)
7. Spirits drinking frequency (days/week)	0 days	139 (36.8%)	8. Shots of spirits per drinking day	0	162 (42.9%)	9. Shots of spirits per week	0	134 (35.4%)
	1–2 days	223 (59.0%)		0.5–1	72 (19.0%)		0.5–2	166 (43.9%)
	3–4 days	10 (2.6%)		2–3	105 (27.8%)		3–5	56 (14.8%)
	5–7 days	6 (1.6%)		>3	38 (10.1%)		6–10	20 (5.3%)
	-	-		-	-		>10	2 (0.5%)
10. Drinking speed	≥1 drink/2–3 h	99 (26.2%)	11. Times drunk in last 6 months	0	172 (45.5%)	12. % of times drunk when drinking	0%	94 (24.9%)
	1 drink/h	127 (33.6%)		1–2	98 (25.9%)		1–20%	103 (27.2%)
	2 drinks/h	105 (27.8%)		3–5	48 (12.7%)		21–50%	106 (28.0%)
	≥3 drinks/h	47 (12.4%)		6–10	16 (4.2%)		51–80%	62 (16.4%)
	-	-		>10	32 (8.5%)		81–100%	13 (3.4%)

**Table 3:** Correlations between the AUQ indices and the scales administered for convergent validity.

AUQ Indices		Binge Eating Scale	Seven Domains Addiction Scales						
			Separation Anxiety	Affect Dysregulation	Somatoform and Psychological Dissociation	Childhood Traumatic Experiences	Impulse Dyscontrol	Compulsive Behaviour and Ritualization	Obsessive Thoughts
Intake Score	*r*	**0.300**	**0.161**	**0.186**	**0.228**	0.090	**0.262**	**0.180**	**0.136**
	*p*	<0.001	0.002	<0.001	<0.001	0.080	<0.001	<0.001	0.008
Binge Drinking Score	*r*	**0.351**	**0.186**	**0.217**	**0.232**	0.085	**0.291**	**0.197**	**0.172**
	*p*	<0.001	<0.001	<0.001	<0.001	0.099	<0.001	<0.001	<0.001

Note. Bolded coefficients are significant at the 0.01 level (two-tailed).

## Data Availability

Data presented in this study are available on request from the corresponding authors.

## Appendix A. Alcohol Use Questionnaire (AUQ)—Italian Version

Le seguenti domande riguardano il tuo uso abituale di vari tipi di bevande alcoliche. Per favore, nel rispondere considera il tuo consumo di alcol negli ultimi sei mesi e prenditi il tuo tempo per dare una risposta accurata a ciascuna domanda.

1. Quanti giorni alla settimana bevi vino o qualsiasi prodotto della tipologia dei vini, ad esempio Sherry, Porto, Martini (almeno un bicchiere piccolo)? _______________________________________ Per favore, indica il/i marchio/i che consumi di solito ________________________________________
2. Nei giorni in cui bevi vino (o simili), quanti bicchieri (misura da pub) bevi? _______________ se non sei sicuro, stima il numero di bottiglie o parti di una bottiglia ___________________________
3. Quanti bicchieri (misura da pub) di vino bevi in una settimana, in totale? ___________________
4. Quanti giorni alla settimana bevi birra o sidro (almeno mezza pinta)? ___________________ Per favore, indica il marchio che consumi di solito (ad es. Carlsberg Special, White Lightning ecc.) ______________________________________________________________________________________
5. Nei giorni in cui bevi birra/sidro, quante pinte bevi di solito? ____________________________
6. Quante pinte di birra/sidro bevi in una settimana, in totale? _____________________________
7. Quanti giorni alla settimana bevi superalcolici (Whisky, Vodka, Gin, Rum ecc.—ma non birra o vino)? ______________________________________________________________________________ Per favore, indica il/i marchio/i che consumi di solito (ad es. Smirnoff, Blue Label etc.) ______________________________________________________________________________________
8. Nei giorni in cui bevi alcolici, quanti shottini (misura da pub) bevi di solito? ______________ in caso di dubbi, stima il numero di bottiglie o parti di una bottiglia ____________________________
9. Quanti bicchieri di superalcolici bevi in una settimana, in totale? __________________________
10. Quando bevi, quanto velocemente bevi? (Qui, una bevanda può essere un bicchiere di vino, una pinta di birra o un shottino di liquori, semplici o misti). Per favore clicca sulla la risposta corretta:
  □ Bevande all’ora: 7+
  □ Bevande all’ora: 6
  □ Bevande all’ora: 5
  □ Bevande all’ora: 4
  □ Bevande all’ora: 3
  □ Bevande all’ora: 2
  □ Bevande all’ora: 1
  □ 1 bevanda in 2 ore
  □ 1 bevanda in 3 o più ore
11. Quante volte ti sei ubriacato negli ultimi 6 mesi? Per “ubriaco” intendiamo perdita di coordinazione, nausea e/o incapacità di parlare chiaramente _______________________________________
12. Qual è la percentuale delle volte che ti ubriachi quando bevi? ____________________________
